# Highly Sensitive and Selective Colorimetric Sensor of Mercury (II) Based on Layer–by–Layer Deposition of Gold/Silver Bimetallic Nanoparticles

**DOI:** 10.3390/molecules25194443

**Published:** 2020-09-27

**Authors:** Arjnarong Mathaweesansurn, Naratip Vittayakorn, Ekarat Detsri

**Affiliations:** 1 Department of Chemistry, Faculty of Science, King Mongkut′s Institute of Technology Ladkrabang, Bangkok 10520, Thailand; naratip.vi@Kmitl.ac.th; 2 Applied Analytical Chemistry Research Unit, Faculty of Science, King Mongkut′s Institute of Technology Ladkrabang, Bangkok 10520, Thailand; 3 Advanced Materials Research Unit, Faculty of Science, King Mongkut′s Institute of Technology Ladkrabang, Bangkok 10520, Thailand; 4 Integrated Applied Chemistry Research Unit, Faculty of Science, King Mongkut′s Institute of Technology Ladkrabang, Bangkok 10520, Thailand

**Keywords:** colorimetric sensor, mercury (II), layer–by–layer deposition technique, bimetallic nanoparticles

## Abstract

A new colorimetric sensor based on gold/silver bimetallic nanoparticles (Au–Ag BNPs) for the sensitive and selective detection of mercury (II) was developed. Gold nanoparticles (AuNPs) were synthesized by Turkevich method. The surface modification of AuNPs was modified by the layer–by–layer technique using poly(diallyl dimethylammonium chloride) which provided positively charged of AuNPs. Negatively charged silver nanoparticles (AgNPs) were synthesized by chemical reduction using poly(4–styrenesulfonic acid–co–maleic acid) as the stabilizing agent. The layer–by–layer assembly deposition technique was used to prepare Au–Ag BNPs of positively and negatively charged of AuNPs and AgNPs, respectively. The synthesized Au–Ag BNPs were characterized by a UV-visible spectrophotometer, zeta potential analyzer, FT–IR, TEM, XRD, and EDX. The Au–Ag BNPs sensor was able to detect mercury (II) in aqueous solution, visibly changing from brownish–orange to purple. The linear relationships of the UV-visible spectrometry demonstrate that the Au–Ag BNPs-based colorimetric sensor can be used for the quantitative analysis of mercury (II) in the range of 0.5–80 mg L^−1^, with the correlation coefficient, r^2^ = 0.9818. The limit of detection (LOD) of mercury (II) was found to be 0.526 + 0.001 mg L^−1^. The BNPs is also verified to have a good practical applicability for mercury (II) detection in the real samples.

## 1. Introduction

Mercury (II) is a very toxic heavy metal, strongly harmful to living things. Natural water can be contaminated by it through industrial waste. Its specific biogeochemical cycling allows bioaccumulation in the food chain leading to serious consequences for animals and humans. Its toxicity depends on a form of mercury and consequently on its ability to accumulate in the environment. Mercury (II) is the main species found in water due to its high solubility [1]. One effect of mercury (II) is well known, namely Minamata syndrome, where the central nervous system, DNA, and mitosis are damaged [2]. Because of these hazards, monitoring of mercury (II) in natural resources is very important.

Many analytical methods have been reported for determining mercury (II) in a variety of samples. Mercury speciation analysis employes commonly used separation techniques, for example gas chromatography (GC) [3], liquid chromatography (LC) [4,5], and capillary electrophoresis (CE) [6], coupled with a very sensitive techniques, including atomic fluorescence spectrometry (AFS) [7] and atomic absorption spectrometry (AAS) [8]. Other methods, including inductively coupled plasma mass spectrometry (ICP-MS) [9], colorimetry [10,11], inductively coupled plasma optical emission spectrometry (ICP−OES) [12], and microwave-induced plasma optical emission spectrometry (MIP−OES) [13] have also been used for the trace analysis of mercury (II). Although these methods are highly sensitive and selective, they generally require complicated sample preparation, expensive instruments, and professional technicians.

Nowadays, metal nanoparticles have been used as chemical sensors, due to their unique physical and chemical properties – different from those of the bulk state [14]. Among these metal nanoparticles, gold nanoparticles (AuNPs) are probably the most prominent metal nanoparticles due to their chemical inertness and easy surface modification. The surface functionalization of AuNPs can be simply accomplished by adding suitable organic molecules into the reaction mixture or directly the AuNPs colloidal solution. These functionalized AuNPs have been used as optical sensors for chemical or biological analyses [15,16,17]. Tolessa et al. used thioglycolic acid functionalized AuNP as a single-drop headspace colorimetric nanosensor for the determination of mercury (II) in environmental waters. This method was sensitive and selective [18]. Chemical sensors based on silver nanoparticles (AgNPs) have been used because of easy visual observation, simple preparation, and low cost [19,20,21]. Using AgNPs optical properties, devices for colorimetric detection based on AgNPs have been extensively used [22,23,24]. Chen et al. developed a method for the colorimetric detection of mercury (II) based on the aggregation of mercury (II)-coated triangular silver nanoplates with the assistance of two lysine amino groups [25]. Although these methods provided good sensitivity and selectivity, they use complicated procedures.

In this work, the prepared gold/silver bimetallic nanoparticles (Au–Ag BNPs) combines the outstanding optical and chemical properties of both AuNPs and AgNP. We developed a simple procedure to synthesize Au–Ag BNPs, using a layer–by–layer technique. The as–prepared BNPs were characterized using a UV-visible spectrophotometer, zeta potential analyzer, FT–IR, TEM, XRD, and EDX. Consequently, the application of the BNPs to colorimetric detection of mercury (II) in water samples was also investigated.

## 2. Results and Discussion

### 2.1. Effect of Concentration of PDADMAC on the Surface Modification of AuNPs

**Figure 1:**
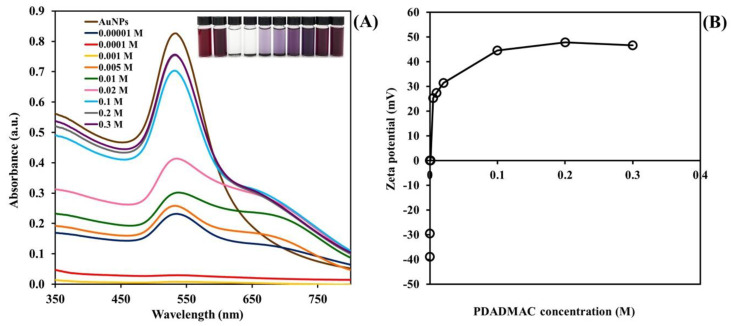
(**A**) Absorption spectra of Au–Ag BNPs and (**B**) Zeta potential of Au–Ag BNPs at different concentrations of PDADMAC.

Poly(diallyl dimethyl ammonium chloride) (PDADMAC) was used as the surface modifying agent for AuNPs in order to generate the positively charges on the surface of AuNPs. Results in Figure 1A show that, when the PDADMAC concentrations are increased (0.01–1.0 mmol L^−1^), the absorbance readings at 533 nm are decreased. The color of solution was changed from red wine to dark purple and colorless. This effect is promoted due to electrostatic interaction between positively charged quaternary ammonium groups of PDADMAC and negatively charged carboxylate on the surface of AuNPs resulting in surface charge neutralization and this event is often associated with the aggregation of AuNPs. At higher concentrations, the positively charged PDADMAC on the surface of AuNPs were increased. This can prevent the assembly of AuNPs and these results in the purple color solution [26]. Moreover, the zeta potential (Figure 1B) of PDADMAC–AuNPs are increased when concentrations of PDADMAC are increased. In this work, 0.1 mol L^−1^ of PDADMAC was chosen as the appropriate concentration.

### 2.2. Characterization of the Synthesized Au–Ag Bimetallic Nanoparticles

**Figure 2:**
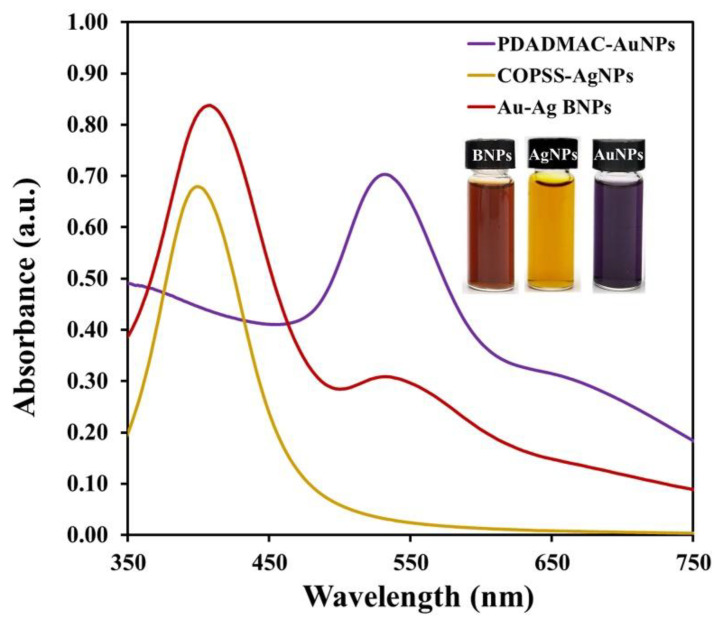
Absorption spectra of PDADMAC–AuNPS, COPSS–AgNPS and the as–prepared Au–Ag BNPs.

**Figure 3:**
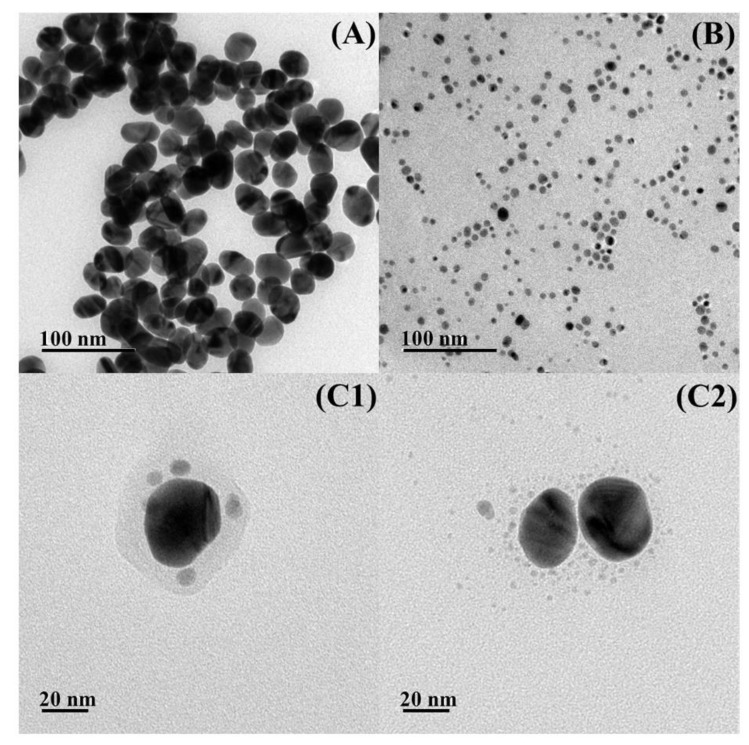
TEM images of (**A**) PDADMAC–AuNPS, (**B**) COPSS–AgNPS and (**C**) the as–prepared Au–Ag BNPs.

Au–Ag BNPs colorimetric sensor was synthesized according to a very simple layer–by–layer method. Firstly, the AuNPs and AgNPs were synthesized using chemical reduction method. The synthesized AuNPs were modified a surface by PDADMAC to generate the positively charges on the surface where the COPSS–AgNPs were deposited. The PDADMAC–AuNPs were then mixed with COPSS–AgNPs. Finally, the Au–Ag BNPs were obtained. The as–synthesized Au–Ag BNPs colloidal solution has an orange–brown color characteristic. Results in Figure 2 show that absorption spectra of Au–Ag BNPs were located at wavelength of 404 nm and 531 nm, which were attributed to the surface plasmon resonance of AuNPs (531 nm) and AgNPs (404 nm). The particle morphologies and size of the as-synthesized Au–Ag BNPs were investigated using TEM analysis. As shown in Figure 3, the spherical–shaped PDADMAC–AuNPs and COPSS–AgNPs are found average particle size of 28.0 ± 1.4 nm and 6.3 ± 0.8 nm (Figure 3A,B). From the TEM images in Figure 3C1,C2, the COPSS–AgNPs were deposited onto the surface of PDADMAC–AuNPs. It is evident that the synthesis of Au–Ag BNPs using the layer–by–layer technique was successful.

**Figure 4:**
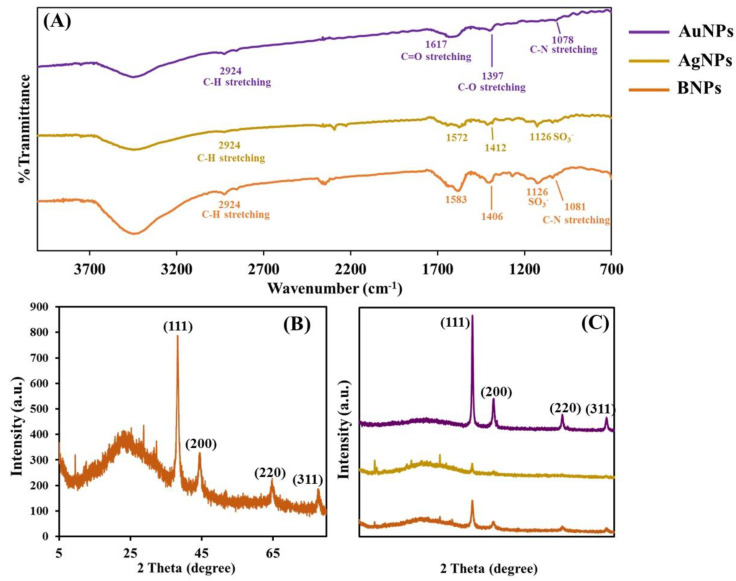
(**A**) the FT–IR spectra of the Au–Ag BNPs compared with the PDADMAC–AuNPs and COPSS–AgNPS, (**B**) The XRD pattern of the as-prepared Au–Ag BNPs and (**C**) The XRD pattern of the as-prepared Au–Ag BNPs compared with the PDADMAC–AuNPs and COPSS–AgNPS.

To confirm the successful formation of the Au–Ag BNPs, FT–IR spectra were used to identify the functional groups and XRD diffraction patterns used to measure the crystallographic structure of the Au–Ag BNPs. The FT–IR spectra of PDADMAC–AuNPs, COPSS–AgNPS and Au–Ag BNPs can be seen in Figure 4A. the FT–IR spectra of the Au–Ag BNPs are similar to that of the PDADMAC–AuNPs and COPSS–AgNPS, where the characteristic C–H stretching bands of PDADMAC–AuNPs and C=O stretching bands of COPSS–AgNPS emerge at 2924 cm^−1^ and 1583 cm^−1^, respectively. The XRD pattern of the synthesized Au–Ag BNPs is illustrated in Figure 4B. Four distinct facets at 2θ value of 38.25°, 44.44°, 64.72°, and 77.65° corresponding to Miller indices (111), (200), (220) and (311) planes of face–centered cubic (FCC) structure of Au–Ag BNPs were obtained, which are similar to that of the AuNPs and AgNPs (Figure 4C). For reference, the prominent peak at 25° of 2θ value was found to be the amorphous characteristic of glass slide substrate. In addition, the EDX spectrometer was also used for element identification. From the EDX spectrum, the peaks of Au and Ag were found (Supplementary Figure S1). These measurements confirmed that the Au–Ag BNPs were successfully synthesized.

### 2.3. Colorimetric Assay of Mercury (II) Detection

**Figure 5:**
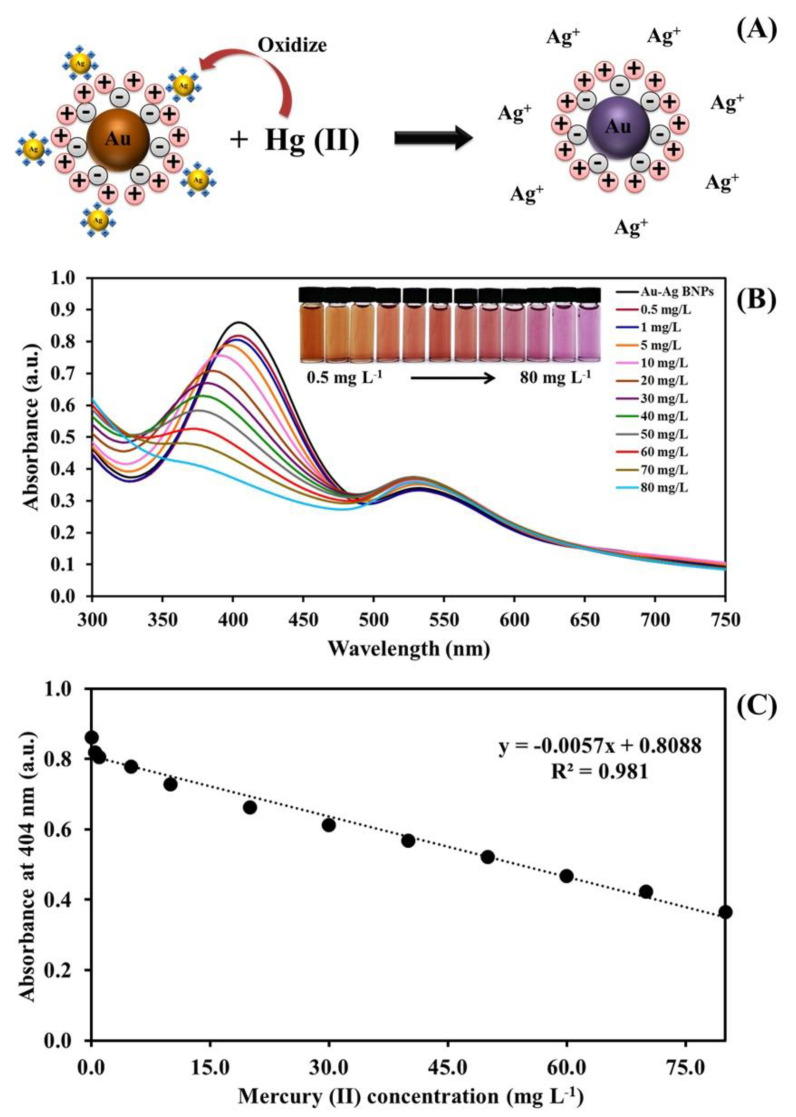
(**A**) The mechanism for colorimetric response of the Au-Ag BNPs to mercury (II), (**B**) Absorption spectra of the Au–Ag BNPs with various standard mercury (II) concentrations and (**C**) linear calibration plot between the absorbance at 404 nm and the standard mercury (II) concentrations.

Following the Au–Ag BNPs synthesis, a colorimetric detection was performed for determination of mercury (II). The detection mechanism of the reaction between the Au–Ag BNPs and mercury (II) is proposed as depicted in Figure 5A. When the mercury (II) was added to the Au–Ag BNPs colloidal solution, oxidation of Ag^0^ by mercury (II) to become Ag^+^ is occurred because the reduction potential of mercury (II) (+0.91 V) is higher than Ag^0^ (+0.80 V) [27]. It results in decreasing in the absorbance at 403 nm (Figure 5B). The colors of the solutions gradually change from orange–brown color to purple when the standard mercury (II) concentrations increase. The linear response of the Au–Ag BNPs to the standard mercury (II) concentration up to 80 mg L^−1^ is also achieved (Figure 5C). Moreover, the zeta potential of Au–Ag BNPs increased significantly from −50.1 mV to −44.0 mV, −33.3 and +33.8 upon the addition of 5.0 mg L^−1^, 40.0 mg L^−1^ and 80.0 mg L^−1^ mercury (II), respectively, which also clearly indicated that Ag^0^ was oxidized and resulting in decreasing in negative charges of COPSS–AgNPs on the Au–Ag BNPs. On the other hand, the positive charges of PDADMAC–AuNPs were appeared (Supplementary Table S1). Therefore, the results revealed that the novel colorimetric Au–Ag BNPs sensor was successfully developed for the determination of mercury (II).

### 2.4. Optimization of the Sensing Conditions

Parameters which affected evaluation of the optical characteristics of Au–Ag BNPs as a mercury (II) sensor, were the incubation time, pH, salt type (NaCl, Na_2_SO_4_, and Na_3_PO_4_) and salt concentration, were investigated.

**Figure 6:**
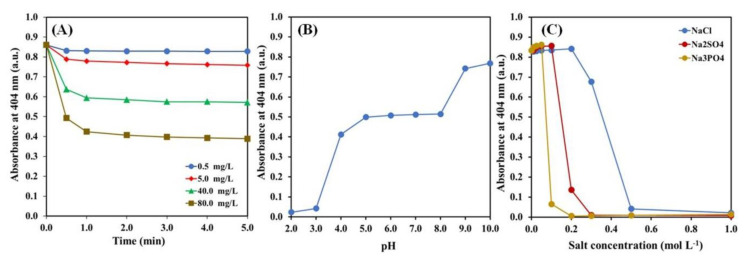
Effect of different parameters on the detection of mercury (II) using the Au–Ag BNPs: (**A**) incubation time, (**B**) pH of the reaction media and (**C**) salt type (NaCl, Na_2_SO_4_ and Na_3_PO_4_) and salt concentration.

Effect of incubation time on colorimetric detection of mercury (II) was investigated from 30 s to 5.0 min. The results in Figure 6A reveal that the absorbance at the wavelength 403 nm of Au–Ag BNPs decreased as the reaction times are prolonged and it reached a relatively constant at 2 min after the addition of high concentration of mercury (II) (80.0 mg L^−1^). This time is therefore chosen as the suitable incubation time.

To investigate the effect of the pH on the detection of mercury (II), the Au–Ag BNPs colloidal solutions were adjusted to various pH (2.0–8.0) using nitric acid or sodium hydroxide. Results in Figure 6B show that the absorbance at the wavelength 403 nm of Au–Ag BNPs is very low when the pH value was less than 3.0, the Au–Ag BNPs was unstable and precipitated. This is because the carboxylate functional group of COPSS is protonated [28]. At the pH range from 4.0 to 8.0, it can exert a great effect on –COOH group of COPSS and Au–Ag BNPs can be more stable [29]. In this work, the pH value of 7.0 is therefore considered as the suitable pH.

Finally, the effect of salt type (NaCl, Na_2_SO_4_ and Na_3_PO_4_) and salt concentration were investigated (Figure 6C). The SO_4_^2−^ and PO_4_^3−^ ions affected the lower of the electrostatic repulsion between Au-Ag BNPs and leading to the aggregation of Au-Ag BNPs. The Au-Ag BNPs were precipitated quickly when the di– or tri– valent salt added. For the Cl^-^ ion, the slow precipitation of Au–Ag BNPs was observed at low NaCl concentration. NaCl could screen the Au–Ag BNPs surface, but the hydrophobic property was still higher than the London–van der Waals force of Au–Ag BNPs [21]. Therefore the high stability was shown with the higher concentration added.

### 2.5. Selectivity of Mercury (II) Detection

**Figure 7:**
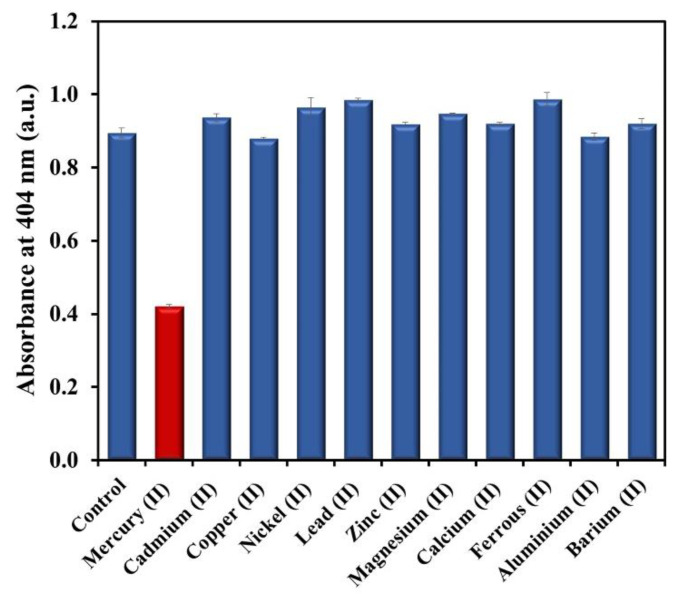
Selectivity study for 80 mg L^−1^ mercury (II) in the presence of interfering species with the concentration 500–fold higher than those of analytes.

The selectivity of this assay was evaluated under the optimized conditions using other metal ions (Cd^2+^, Cu^2+^, Ni^2+^, Pb^2+^, Zn^2+^, Mg^2+^, Ca^2+^, Fe^2+^, Al^2+^ and Ba^2+^). The concentration of interferences was 500 times higher than that of mercury (II) (80 mg L^−1^). Figure 7 clearly illustrates that only mercury (II) caused an absorbance decrease at wavelength 404 nm. This result demonstrated that the Au–Ag BNPs were more selective to mercury (II) than the other metal ions.

### 2.6. Analytical Performances of Gold/Silver Bimetallic Nanoparticles for Mercury (II) Assay

To evaluate the performance of Au–Ag BNPs for determination of mercury (II), linearity, limit of detection (LOD), limit of quantification (LOQ), accuracy, and precision were investigated. Under the optimal conditions, A linear relationship between the absorbance at wavelength 404 nm and the concentration of mercury (II) was found in the range of 0.5–80 mg L^−1^ with good linearity (r^2^ = 0.981). The LOD (3SD of blank/slope of calibration) and the LOQ (10SD of blank/slope of calibration) were 0.53 and 1.75 mg L^−1^, respectively. Such a concentration level was lower to those achieved by the other mercury (II) assays, as shown in Table 1. The recovery was found in the range of 98.2–106.1%. The reproducibility of this assay was studied by a series of ten repetitive measurements of 0.5, 25.0, 50.0 and 75.0 mg L^−1^ mercury (II), corresponding to the relative standard deviation (RSD) of 1.59%, 0.39 %, 1.43% and 0.09%, respectively. This means the proposed method provides high accuracy and precision.

### 2.7. Application for the Detection of Mercury (II) in Aqueous Media

To demonstrate the applicability of the Au–Ag BNPs for analysis of real sample, they were applied to river water for colorimetric determination of mercury (II). After sample collection from the rivers near Ladkrabang industrial estate, they were filtered through a 0.22-µm membrane filter prior to analysis. In addition, different concentrations of standard mercury (II) were spiked into the water sample to ensure the reliability of the results. The results determined by the proposed method were compared to the results obtained using the validating cold vapor-atomic fluorescence spectrometric method. The results of the samples were summarized in Table 2. The results in Table 2 confirm that the compared methods are not significant different based on the statistical paired *t*–test (*t*_stat_ = 0.21, *t*_crit_ = 2.26), indicating that the developed method can be applied for mercury (II) detection in a water sample.

## 3. Materials and Methods

### 3.1. Chemicals and Reagents

Gold (III) chloride trihydrate and sodium citrate tribasic dihydrate (purchased from Sigma-Aldrich, USA) were used to synthesize gold nanoparticles. Poly(diallyl dimethyl ammonium chloride) (PDADMAC) (Sigma-Aldrich, St. Louis, Missouri, USA) was exploited as a surface modifying agent. Silver (Carlo Erba, Lombardia, Italy) was used for synthesis of silver nanoparticle. Sodium borohydride and Poly(4-Styrenesulfonic acid-co-maleic acid) (COPSS) were purchased from Sigma-Aldrich and were exploited as reducing agent and stabilizing agent, respectively. A standard stock mercury (II) solution (100 mg L^−1^) was prepared by dissolving 0.0100 g of mercury (II) chloride powder (Sigma-Aldrich) in 100.0 mL of water. The working standards solution were prepared by appropriate dilution of the stock solution. Deionized-distilled water (18 MΩ cm^−1^) ((Milli-Q^®^, Millipore system) was used throughout. All glassware used were immersed in 10% nitric acid for overnight and rinsed with deionized, distilled water. Nitric acid and sodium hydroxide (Carlo Erba) were used to adjust pH.

### 3.2. Synthesis of Gold/Silver Bimetallic Nanoparticles

**Scheme 1:**
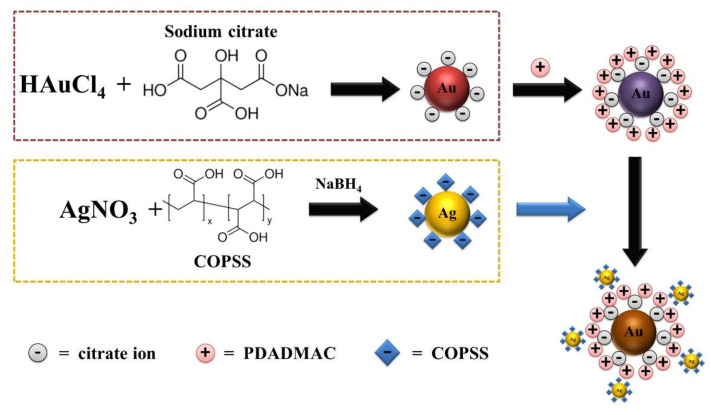
Gold/silver bimetallic nanoparticles synthesized procedures.

Au–Ag BNPs synthesized procedures were illustrated in Scheme 1. Briefly, first, to prepare positively charged AuNPs, 18.0 mL of 1.0 mmol L^−1^ gold (III) chloride trihydrate solution was heated to 80 °C and then aliquot of 0.5 mL of sodium citrate was rapidly transferred under vigorous stirring for 15 min. During this time, the initial yellow color of the solution was changed to red wine color. An aliquot of 5.0 mL of AuNPs solution was mixed with 0.1 mL of 0.1 mol L^−1^ PDADMAC. The mixed solution was then incubated at room temperature for 3 days. The color of PDADMAC–AuNPs colloidal solution turned to purple. The negatively charged silver nanoparticles were prepared by chemical reduction method. The 1.0 mL of 0.1 mol L^−1^ silver nitrate solution and 5.0 mL of 0.1 mol L^−1^ COPSS were added into 74.0 mL of DI water. Then, 20.0 mL of 50 mmol L^−1^ NaBH_4_ was rapidly added to the mixture solution under stirring for 5 min. The color of solution changed to yellow.

Next, Au–Ag BNPs were synthesized by layer–by–layer deposition technique as BNPs were constructed by the deposition of negatively charged silver nanoparticles onto the surface of positively charged AuNPs. Briefly, the 2.0 mL of AgNPs colloidal solution was added into the 2.0 mL of PDADMAC–AuNPs colloidal solution. The mixed solution was then incubated at room temperature for 24 h. A brown color solution of Au–Ag BNPs was obtained. Finally, the solution was stored at 4.0 °C in the refrigerator for further use.

### 3.3. Characterizations

The optical properties of Au–Ag BNPs were characterized using UV1800 UV-visible spectrophotometer (Shimadzu Co. Ltd., Beijing, China) with a matched pair of 10 mm quartz cuvette. The prepared functional groups of Au–Ag BNPs was characterized by a Tracer–100 Fourier transform–infrared (FT–IR) spectrometer (Shimadzu Co. Ltd, Kyoto, Japan). The zeta potential analyzer (surface charges) was measured using Zeta sizer Nano, ZS with 633 nm Helium–Neon laser (Malvern instrument, Worcestershire, England). Transmission electron microscope (TEM, JEM–2001 model, JEOL Co., Ltd., Tokyo, Japan) was used to evaluate the morphology and size of Au–Ag BNPs. The crystal structure identification was performed by XRD–6100 X–ray diffractometer (Shimadzu Co. Ltd., China). The element identification was evaluated using X–MaxN energy dispersive X-ray spectrometer (EDX, Oxford instrument, London, UK).

### 3.4. Colorimetric Detection of Mercury (II)

The 1.5 mL of the synthesized BNPs solution was 4.5-fold diluted with DI water. An aliquot of 2.0 mL of the diluted BNPs solution was pipetted into beaker, followed by adding 1.0 mL of different concentrations from 0.5 mg L^−1^ to 80.0 mg L^−1^ of mercury (II). The mixture solutions were incubated for 2 min at room temperature. The surface plasmon band of the solution was recorded from 300 to 800 nm using a UV-visible spectrophotometer. Calibration plot between the absorbance at central maximum wavelength (λ_max_) 404 nm against the standard mercury (II) concentrations was exploited for the quantification of mercury (II) in sample solution.

### 3.5. Colorimetric Detection of Mercury (II) Selectivity of Mercury (II) Detection

The 1.5 mL of the synthesized BNPs solution was 4.5-fold diluted with DI water. An aliquot of 2.0 mL of the diluted BNPs solution was pipetted into beaker, followed by adding 1.0 mL of the interference (aluminium sulfate, barium sulfate, cadmium sulfate, calcium nitrate, copper nitrate, ferrous sulfate, lead nitrate, magnesium nitrate, nickel nitrate, and zinc sulfate). The mixture solutions were incubated for 2 min at room temperature. The surface plasmon band of the solution was recorded from 300 to 800 nm using a UV-visible spectrophotometer.

### 3.6. Application for the Detection of Mercury (II) in Aqueous Media

The proposed Au–Ag BNPs colorimetric sensor probe was applied for mercury (II) determination in water sample. The water samples were collected from river in Bangkok, Thailand. Prior to analysis, the water sample was prepared by filtering through a 0.22 µm nylon membrane. The 1.0 mL of water samples was subsequently mixed with the 1.5 mL of the prepared BNPs solution and incubated for 3 min at room temperature. The surface plasmon band was monitored from 300 to 800 nm using a UV-visible spectrophotometer. To ensure the applicability and reliability of the developed method, cold vapor atomic fluorescence spectrometry (CV–AFS) was also exploited as the validation technique.

## 4. Conclusions

A new colorimetric sensor based on Au–Ag bimetallic nanoparticles for the sensitive and selective detection of mercury (II) was developed. The synthesis of stable Au–Ag BNPs using the layer–by–layer deposition technique was easy. the quantitative determination of mercury (II) in natural water samples was investigated. The orange–brown color of Au–Ag BNPs colloidal solution changed to purple in accordance with the mercury (II) level added as the oxidation of Au–Ag BNPs by mercury (II) lead to decreasing of the absorbance at wavelength 404 nm. The parameters affecting the detection, including incubation time, pH, salt type (NaCl, Na_2_SO_4_, and Na_3_PO_4_), and salt concentration were explored. Under the optimal conditions, the detection of mercury (II) using Au–Ag BNPs was found in the concentration range of 0.5–80 mg L^−1^ with the limit of detection of 0.53 mg L^−1^. The results revealed that the novel colorimetric Au–Ag BNPs sensor was successfully developed. The method also provided highly sensitive, selective, simple, and rapid for the determination of mercury (II) in natural water samples.

## Figures and Tables

**Table 1:** Comparison of different methods for determination of mercury (II)**.**

Colorimetric Probe	LOD (µM)	Linear Range (µM)	Ref.
Ultrasmall Ce^3+^ and Tb^3+^ doped SrF_2_ nanocrystals capped with glutathione	20.00	1–10	[30]
Counterion exchange of cationic polydiacetylene	8.30	0–65	[31]
Dichlorofluorescein—coumarin conjugate	4.30	0–100	[32]
Rhodamine derivative functionalized chitosan	3.42	0–6	[33]
Au-Ag Bimetallic nanoparticle	2.64 (0.53 mg L^−1^)	0–398	This work

**Table 2:** Determination of mercury (II) in water samples using the Au-Ag BNPs colorimetric sensor and CV–AFS method.

Sample	This Work (mg L^−1^)			CV–AFS (mg ^L−1^)		
	Original*	Added	Found **	Original*	Added	Found **
S1	n.d.	5.0	5.6 ± 0.2	n.d.	5.0	5.1 ± 0.2
	n.d.	15.0	16.3 ± 0.4	n.d.	15.0	16.0 ± 0.1
S2	n.d.	5.0	6.3 ± 0.1	n.d.	5.0	5.9 ± 0.3
	n.d.	15.0	15.6 ± 0.2	n.d.	15.0	15.8 ± 03
S3	n.d.	5.0	6.1 ± 0.3	n.d.	5.0	6.6 ± 0.2
	n.d.	15.0	16.6 ± 1.1	n.d.	15.0	16.2 ± 0.4
S4	n.d.	5.0	5.9 ± 0.5	n.d.	5.0	7.2 ± 0.7
	n.d.	15.0	16.4 ±0.3	n.d.	15.0	16.6 ± 0.2
S5	n.d.	5.0	6.4 ± 0.2	n.d.	5.0	6.9 ± 0.2
	n.d.	15.0	16.2 ± 0.8	n.d.	15.0	16.7 ± 0.4

Note: * n.d. = non detectable, ** mean + SD (n = 3).

## Supplementary Materials

The following are available online, Figure S1: the EDX spectrum of the as-prepared Au–Ag BNPs., Table S1: Zeta potential of Au-Ag BNPs used as sensor for determination of mercury (II).
