# Supplementary material for: Highly Sensitive and Selective Colorimetric Sensor of Mercury (II) Based on Layer–by–Layer Deposition of Gold/Silver Bimetallic Nanoparticles
Source: Molecules. 2020 Sep 27;25(19):4443. doi: 10.3390/molecules25194443 (PMC7583855; doi:10.3390/molecules25194443)
Supplement: Supplementary file 1 [file molecules-25-04443-s001.pdf]

Supplementary Material

Table S1. Zeta potential of Au-Ag BNPs used as sensor for determination of mercury (II).

Mercury concentration (mg L^{-1})	Zeta Potential (mV)
0	−50.14 ± 1.21
5	−44.07 ± 2.39
40	−33.39 ± 1.82
80	33.80 ± 1.95

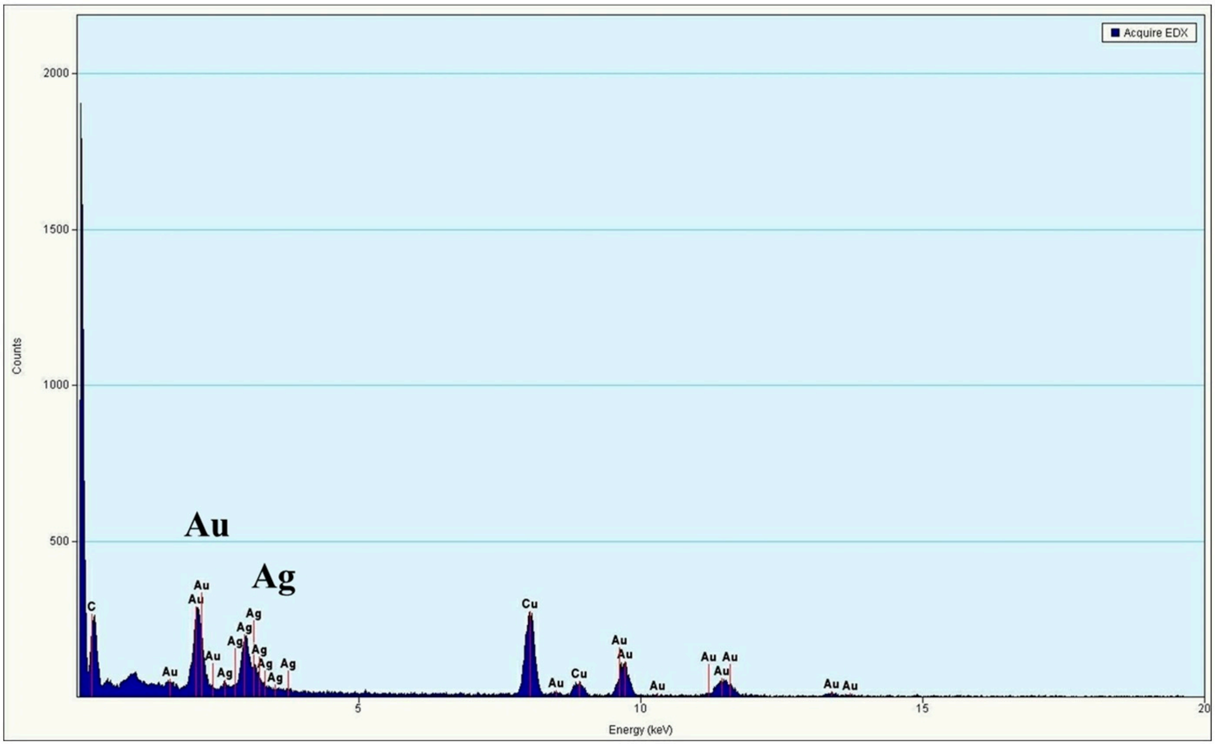

Figure S1. the EDX spectrum of the as-prepared Au–Ag BNPs.
